# Treatment of Recurrent Disc Herniation: A Systematic Review

**DOI:** 10.7759/cureus.622

**Published:** 2016-05-23

**Authors:** Doniel Drazin, Beatrice Ugiliweneza, Lutfi Al-Khouja, Dongyan Yang, Patrick Johnson, Terrence Kim, Maxwell Boakye

**Affiliations:** 1 Department of Neurosurgery, Cedars-Sinai Medical Center; 2 Department of Neurosurgery, University of Louisville; 3 Department of Epidemiology and Population Health, University of Louisville; 4 Deparment of Orthopedics, Cedars-Sinai Medical Center

**Keywords:** recurrent disc herniation, recurrent lumbar disc herniation, spine, spinal fusion, revision fusion, minimally invasive lumbar fusion, interbody fusion, back pain

## Abstract

Intervertebral disc herniation is one of the most common causes of back and extremity pain. The most commonly used surgical treatment is lumbar discectomy. About 0.5-25% go on to develop recurrent disc herniation (rDH) after a successful first discectomy. Currently, there aren’t any guidelines to assist surgeons in determining which approach is most appropriate to treat rDH. A recent survey showed significant heterogeneity among surgeons regarding treatment options for rDH. It remains unclear which methods lead to better outcomes, as there are no comparative studies with a sufficient level of evidence. In this study, we aimed to perform a systematic review to compare treatment options for rDH and determine if one intervention provides better outcomes than the other; more specifically, whether outcome differences exist between discectomy alone and discectomy with fusion.

We applied the PICOS (participants, intervention, comparison, outcome, study design) format to develop this systematic review through PubMed. Twenty-seven papers from 1978-2014 met our inclusion criteria and were included in the analysis. Nine papers reported outcomes after discectomy and seven of them showed good or excellent outcomes (70.60%-89%). Ten papers reported on minimally invasive discectomy. The percent change in visual analog scale (VAS) ranged from -50.77% to -86.57%, indicating an overall pain reduction. Four studies out of the ten reported good or excellent outcomes (81% to 90.2%). Three studies looked at posterolateral fusion. Three studies analyzed posterior lumbar interbody fusion. For one study, we found the VAS percentage change to be -46.02%. All reported good to excellent outcomes. Six studies evaluated the transforaminal lumbar interbody fusion. All reported improvement in pain. Four used VAS, and we found the percent change to be -54% to -86.5%. The other two used the Japanese Orthopedic Association (JOA) score, and we found the percent change to be 68.3% to 93.3%.

We did not find enough evidence to support any significant difference in outcomes between discectomy alone and discectomy with fusion. The limitation of our study includes the lack of standardized outcomes reporting in the literature. However, reviewing the selected articles shows that fusion may have a greater improvement in pain compared to reoperation without fusion. Nonetheless, our study shows that further and more in-depth investigation is needed on the of treatment of rDH.

## Introduction

Intervertebral disc herniation is one of the most common causes of back and extremity pain that can eventually require surgical intervention. Many surgical approaches have been utilized to treat disc herniation where the type of surgery is dependent upon the level of herniation, type of herniation, symptomatology, and surgeon preference. The most commonly used surgical method is a lumbar discectomy [1].

Disc reherniation is the most common cause of reoperation after primary disc surgery and is defined as disc herniation occurring at the same level in a patient after a definite pain-free period of at least six months from initial surgery [2]. Rates of recurrent disc herniation (rDH) have been reported to be between 0.5% and 25% [3]. Although there are many theories as to what increases a patient’s chance for reherniation, no one factor has been identified consistently in the literature. Some of these proposed risk factors include obesity, smoking, male gender, diabetes, weightlifting, the size of the annular tear, and type of primary operation [4-17]. Other causes of reoperation include new disc herniation at a different level, epidural fibrosis, adhesive arachnoiditis, spinal stenosis, and segmental instability [18].

Currently, there are no guidelines or significant comparative studies to assist surgeons in determining which approach would be most appropriate to treat rDH. The American Association of Neurologic Surgeons (AANS) 2014 guidelines report low-level evidence to support fusion for rDH and call for further investigations with improved study designs to better address this issue [19]. In the absence of guidelines to approach patients with rDH, there are significant differences in treatment plans among spine surgeons in the United States, which was evaluated in a survey of spine surgeons by Mroz, et al. [20]. Their survey found that a patient’s treatment plan varied based on surgeon experience and operative volume. With the prognosis of repeated back surgery being relatively poor in regards to pain relief and return to work [21], identifying the appropriate treatment for recurrent disc herniation is important to improve prognosis. A recent recommendation by Wang, et al. is to perform a discectomy in patients with rDH and radiculopathy [19]. Fu, et al. reported similar recommendations. Additionally, fusion has been recommended if the patient has associated lumbar instability, radiographic degenerative changes, and/or chronic axial lower back pain [22]. However, a repeat discectomy is generally more difficult due to scar tissue from the primary surgery, and there is an increased the risk of dural tears or nerve injury [23]. Furthermore, using a minimally invasive percutaneous endoscopic method was determined to be effective in decreasing the chance of fusion and bleeding with reoperation in comparison to conventional revision discectomy [23]. A retrospective study by Ambrossi, et al. found a substantial amount of healthcare costs associated with recurrent disc herniation averaging $26,593 per patient to diagnose and manage [24]. All in all, it is still unclear which method has shown to be more effective for reoperation.

There are currently no studies directly comparing the various treatments of rDHs. The goal of this systematic review is to compare the various treatment options for rDH and determine if one intervention provides better outcomes than the others. More specifically, if there is a difference in outcomes from surgery with and without fusion.

The PICOS format is a technique used to help formulate a clinical question and guide the subsequent literature search to provide an evidence-based technique to acquire clinical information from the literature [25-26]. Applying the PICOS format in developing this systematic review, we established the following criteria:

*- Participants:* Adults ≥ 18 with recurrent disc herniation

*- Interventions:* discectomy, minimally invasive surgical (MIS) discectomy, posterolateral fusion (PLF), posterior lumbar interbody fusion (PLIF), transforaminal interbody fusion (TLIF), anterior lumbar interbody fusion (ALIF)

*- Comparisons:* discectomy, MIS discectomy, PLF, PLIF, TLIF, ALIF

*- Outcomes:* any

*- Study Designs:* any

The hope is that this study, with the above criteria, will help to determine the advantages and disadvantages of various interventions to treat rDH.

## Materials and methods

### Literature search

A literature search was performed using PubMed with the search term “recurrent disc herniation” with MeSH terms "intervertebral disc displacement”, “reoperation”, and “recurrence”. The search was performed on June 5, 2015. Studies were excluded if they did not address the treatment of recurrent disc herniation, did not state the specific intervention being studied, did not report validated outcomes of that specific intervention, or did not have an adequate sample size (which was arbitrarily determined to be ≥ 10 patients per study group). No preference was taken to the type of study (prospective, retrospective, etc.), the length of follow-up, or status of publication. Ultimately, we included papers that had covered a specific surgical treatment option for recurrent lumbar disc herniation that reported the outcomes of the intervention from different studies with an adequate sample size. We first reviewed the abstracts of all the articles that populated following the search for inclusion and exclusion criteria. Then, an in-depth review of each individual article was conducted for further inclusion into our analysis.

### Data variables

While reading through each paper, we looked at the type of surgery used, study type, length of follow-up, time spent in the operating room, estimated blood loss, costs associated with re-operation, visual analogue scale ratings (VAS, pre- and postoperatively), Oswestry Disability Index (ODI, pre- and postoperatively), length of stay (LOS), re-operation outcomes, complications with re-operation, and percent with good or excellent outcomes. Percent differences of preoperative and postoperative VAS and ODI were calculated by dividing the difference over the preoperative score:

$Percent Difference VAS or ODI = \frac{Preoperative VAS or ODI - Postoperative VAS or ODI}{Preoperative VAS or ODI} x 100$

$Percent Difference JOA = \frac{Postoperative JOA - Preoperative JOA}{Postoperative JOA} x 100$

In calculating the percent difference in VAS and ODI, we were able to establish an internal control for each study and more accurately present the average changes in subjective and objective outcomes after surgery rather than comparing the raw numbers from each study.

## Results

Using the queries listed above, a search through PubMed resulted in 106 abstracts that met initial screening criteria. Careful analysis of these 106 articles brought us to 27 that fit the inclusion criteria to be part of the analysis. Of note, some of these 27 articles discussed more than one type of surgery. There were eight articles studying repeat surgery with fusion, 17 without fusion, and two studying both. A summary of these papers is listed is listed in Tables 1-2. 

**Table 1:** Treatment of Recurrent Disc Herniation without Spinal Fusion Studies EBL: Estimated Blood Loss; VAS: Visual Analog Scale; ODI: Oswestry Disability Index; LOS: Length of Stay; TLIF: Transforaminal Interbody Fusion; DVT: Deep Vein Thrombosis; PLF: Posterior Lumbar Fusion; JOA: Japanese Orthopedic Association; NR: Not Reported; CSF: Cerebrospinal Fluid; PDTS: Posterior Dynamic Transpedicular Stabilisation; PELD: Percutaneous Endoscopic Lumbar Discectomy; OLM: Open Lumbar Discectomy

	Article	Surgery Type	N (% Female)	Study Type	Average Follow-Up, Months (Range)	OR Time, Minutes (Range)	EBL, mL (Range)	Costs	Percent Change in VAS	Percent Change in ODI	Postop LOS, Days (Range)	Outcomes	Percent Showing Good or Excellent Outcomes	Complications in Repeat Surgery
1	El Shazly, 2013 [27]	Discectomy	15 (46.7%)	Prospective, Randomized, Comparative	38.6 ± 7.73	125.3 ± 25.32	256.7 ± 67.13	$1,520 ± 36.84	+52.17% in JOA score	NR	3.4	Overall, all three methods showed significant improvements postoperatively. Discectomy with fusion was associated with better improvement in pain and less complications. PLF was more cost-effective compared to TLIF	86.70%	Recurrent herniation x1, postop instability x1, postop neurological deficit x2, dural tear x 4
		Discectomy with TLIF	15 (40%)		36.3 ± 8.06	194 ± 25.58	653.3 ± 183.68	$2,776.7 ± 56.27	+70.0% in JOA score	NR	3.5		93.30%	Postop neurological deficit x1, dural tear x2, DVT x1
		Discectomy with PLF	15 (46.7%)		36.1 ± 8.05	186 ± 16.82	660 ± 164.97	$2,186.7 ± 52.33	+60.71% in JOA score	NR	3.3		86.70%	Dural tear x1, superficial wound infection x1
2	Kim, 2012 [37]	Microdiscectomy with CO2 Laser Dissection	21 (42.9%)	Retrospective	30 (9 - 36)	NR	NR	NR	-60.53%	-61.32% (Korean version of ODI)	5.14 (2 - 15)	Significant improvement of pain postoperatively in relation to VAS and ODI	NR	None
3	Ahsan, 2012 [30]	Discectomy	18 (22.%2)	Retrospective	NR (12-48)	141 ± 9	NR	NR	-83.53%	-77.92%	5 (3 - 8)	Results of repeat discectomy comparable to primary surgery	85%	Foot drop x1, dural tear x3, superficial wound infection x1
4	Shin, 2011 [23]	Endoscopic Discectomy	41 (31.7%)	Retrospective	16 (13-42)	37 (25 - 96)	Minimal	NR	Back Pain: -34.48%; Leg Pain: -67.05%	NR	NR	Much improvement in pain without serious neurological deficits or compliations	90.20%	Thecal sac injury with CSF leak x2, transient postoperative dysesthesia x2, recurrence x2,
5	Kaner, 2010 [36]	Microdiscectomy with PDTS	40 (42.5%)	Prospective	41 (24 - 63)	NR	NR	NR	-86.57%	-88.56%	NR	Satisfactory improvement in VAS/ODI scores at 2-year follow-up	NR	Foreign body reaction x1, repeat operation for dynamic system removal and fusion x1
6	Lee, 2009 [39]	PELD	25 (36.0%)	Retrospective	34.0 ± 4.4	45.8 ± 11	NR	NR	Back: -58.57% Leg: -65.48%	-66.40%	0.9 ± 0.5	PELD and OLM both showed favorable outcomes, but PELD with shorter OR time, shorter LOS, and better disc height preservation compared to OLM	NR	1 patient with persistent leg pain underwent repeat operation with OLM; Recurrence in 1 patient
		Open Lumbar Microdiscectomy	29 (24.1%)		34.6 ± 4.6	73.8 ± 25.7			Back: -42.59% Leg: -59.30%	-71.16%	3.8 ± 1.4			Dural tear x2, voiding difficulty and perineal dysesthesia x1; Recurrence in 3 patients
7	Kim, 2009 [38]	Microdiscectomy	14 (21.4%)	Retrospective	56 (36-72)	NR	NR	NR	NR	NR	NR	The surgical outcome of first operation was 79.7% ± 9.3% and of the second operation was 77.8% ± 10.4%	NR	NR
8	Ambrossi, 2009 [24]	Conservative	6	Retrospective	12	NR	NR	$2,315	NR	NR	NR	5 patients underwent a single epidural steroid injection and 4 patients underwent 4 weeks of outpatient physical therapy	NR	NR
		Discectomy	11					$39,836				1 patient without symptom relief after surgery who subsequently underwent fusion		
9	Guo, 2009 [29]	Discectomy by Fenestration	51 (25.5%)	Retrospective	146.8	NR	NR	NR	+64.8% in JOA score	NR	NR	8 patients (15.7%) failed revision open lumbar discectomy by fenestration	70.60%	5 dural tears, 2 nerve root injuries, and 1 deep infection
10	Palma, 2008 [32]	Discectomy	95 (30.5%)	Retrospective	NR	110	NR	NR	NR	NR	NR	Overall longer OR time for reoperation compared to primary surgery (110 vs. 75) and an unsuccessful surgery rate of 2%	89% (compared to 95% after primary operation)	4 dural tears
11	Hoogland, 2008 [35]	Endoscopic Transforaminal Discectomy	262 (29%)	Prospective	24	NR	NR	NR	-66.71% in back pain; -69.14% in leg pain	NR	NR	Average improvement of back pain of 5.71 points and 5.85 points of leg pain on the VAS scale	85.71%	10 patients with complications (3.82%): 3 nerve root irritations and 7 early recurrent herniations
12	TS Fu, 2005 [22]	Discectomy	23	Retrospective	88.7 (60 –134)	100.9 ± 22.8	162.7 ± 106.8	NR	+62.45% in JOA score	NR	4.7 ± 1.4	Intraoperative blood loss, length of surgery, and length of hospitalization were significantly less in patients undergoing discectomy alone than in patients with fusion.	78.3% based on JOA score	3 dural tears
		Discectomy with PLF	18			166.3 ± 26.7	546.7 ± 206.1	NR	+66.02% in JOA score	NR	6.2 ± 1.1		83.3% based on JOA score	1 superficial infection, 2 dural tears, 3 residual donor site pain
13	LY Dai, 2005 [28]	Discectomy	39 (41%)	Retrospective	92	NR	NR	NR	+58.62% in JOA score	NR	NR	The outcomes of repeat discectomy for recurrent disc herniation were satisfactory; 29/39 returned to previous work status or normal daily activities	74.36% with excellent outcomes	7 dural tears
14	Ahn, 2004 [33]	PELD	43 (25.6%)	Retrospective	31 (24-39)	51 (25-100)	NR	NR	-70.41%	NR	NR	The percentage of successful outcomes was 81.4%, whereas the rate of improvement was 95.3%	81.40%	1 with incomplete decompression and was converted to open discectomy; 2 with transient dysesthesia
15	Suk, 2001 [45]	Discectomy	28 (40.0%)	Retrospective	NR	88.9	NR	NR	NR	NR	12.9	Conventional open discectomy as a revision surgery for recurrent lumbar disc herniation showed satisfactory results that were comparable with those of primary discectomy	NR	NR
16	Cinotti, 1998 [34]	Microdiscectomy	26 (31.0%)	Prospective	24 months	NR	NR	NR	UTD	NR	1.5 (1-3)	17 patients were able to return to full employment and 4 were able to return to regular daily activities at same level as prior to primary discectomy	81%	2 dural tears, 1 with postop discitis, 1 with second recurrent herniation
17	Silvers, 1994 [40]	Microdiscectomy	82 (35.0%)	Retrospective	46.8 (< 12-168)	NR	NR	NR	NR	NR	4.7	Patients who presented within one year of primary surgery with same level and same side recurrence had poor outcomes following microdiscectomy	NR	10 dural tears (4 with CSF leakage), 6 wound infections, 2 pseudomeningoceles, 1 wound hematoma
18	Herron, 1994 [31]	Laminectomy and Discectomy	46	Retrospective	54 (12-128)	NR	NR	NR	NR	NR	NR	Satisfactory outcomes in treatment of rLDH without associated spinal instability. Most patients experienced "good" surgical outcomes with >75% relief in back and leg pain	>75% with good surgical outcomes	NR
19	Hou, 2015 [46]	Repeat Microendoscopic Discectomy	25 (52%)	Prospective	36 (12-72)	85 (60-100)	68 (20-100)	NR	Leg Pain: -71.6%	-54.80%	NR	No nerve root or cauda equina injury	96%	Small dural tear x3, Recurrence x1 resulting in fusion

**Table 2:** Treatment of Recurrent Disc Herniation with Spinal Fusion Studies EBL: Estimated Blood Loss; VAS: Visual Analog Scale; ODI: Oswestry Disability Index; LOS: Length of Stay; PLF: Posterior Lumbar Fusion; PLIF: Posterior Lumbar Interbody Fusion; NR: Not Reported; UTI: Urinary Tract Infection; TLIF: Transforaminal Lumbar Interbody Fusion; MIS: Minimally Invasive Surgery; DVT: Deep Vein Thrombosis; JOA: Japanese Orthopedic Association; PSI: Pedicle Screw Instrumentation; UTD: Unable to Determine; PSF: Posterior Spinal Fusion

	Article	Surgery Type	N (% Female)	Study Type	Average Follow-Up, Months (Range)	OR Time, Minutes (Range)	EBL, mL (Range)	Costs	Percent Change in VAS	Percent Change in ODI	Postop LOS, Days (Range)	Outcomes	Percent Showing Good or Excellent Outcomes	Complications in Repeat Surgery
1	Niu, 2005 [41]	PLF/PLIF by Dual Cages	14 (43%)	Prospective	25 (14-36)	230 (150 - 350)	623 (200 - 1,300)	NR	NR	NR	NR	No neurological deficits.	93%	Superficial wound infection x2, UTI x1, wedged disc x1
2	Li, 2015 [48]	TLIF	73 (42%)	Retrospective, Unrandomized, case control	49 (12-85)	105 (70 - 260)	260 (90 - 800)	NR	Leg: -86.5%; Back: -84.9%	-55.70%	8.5	No implant failure. Successful fusion in 92.3%. No permanent neurological deficit	91.80%	Dural laceration x3, transient neuro deficits x5, revision surgery x3
3	Omid-Kashani, 2014 [47]	TLIF	51 (59%)	Retrospective	31.4 (25-50)	NR	NR	NR	Leg: -54%; Back: -55.1%	-61.90%	NR	Fusion rate 100%, no instrument failure	74.60%	Iatrogenic partial L5 root injury x1
4	Niesche, 2014 [43]	MIS-TLIF	14	Retrospective	52 (48 - 59)	140 (95 - 190)	150 (120 - 370)	NR	-56.52%	-64.71%	5 (3 - 7)	Solid radiographic fusion at 24 months; no development of adjacent disc disease	85%	None
		Open-TLIF	19			130 (80 - 190)	380 (350 - 620)	NR	-56.52%	-64.71%	10 (8-14)	Solid radiographic fusion at 24 months; improvement in VAS and ODI not as significant compared to MIS	68.30%	4 revisions due to wound healing disorders, 2 with neurologic deterioration due to radiculopathy
5	Lequin, 2014 [21]	PLIF	26	Retrospective	15.3	NR	NR	NR	-46.02%	NR	NR	85% with subjective improvement after reoperation	46% with good recovery	2 hematomas, 2 dural tears, 4 with increased/new neurologic deficits, 1 superficial wound infection
6	El Shazly, 2013 [27]	Discectomy	15 (46.7%)	Prospective, Randomized, Comparative	38.6 ± 7.73	125.3 ± 25.32	256.7 ± 67.13	$1,520 ± 36.84	+52.17% in JOA score	NR	3.4	Overall, all three methods showed significant improvements postoperatively. Discectomy with fusion was associated with better improvement in pain and less complications. PLF was more cost-effective compared to TLIF	86.70%	Recurrent herniation x1, postop instability x1, postop neurological deficit x2, dural tear x 4
		Discectomy with TLIF	15 (40%)		36.3 ± 8.06	194 ± 25.58	653.3 ± 183.68	$2,776.7 ± 56.27	+70.0% in JOA score	NR	3.5		93.30%	Postop neurological deficit x1, dural tear x2, DVT x1
		Discectomy with PLF	15 (46.7%)		36.1 ± 8.05	186 ± 16.82	660 ± 164.97	$2,186.7 ± 52.33	+60.71% in JOA score	NR	3.3		86.70%	Dural tear x1, superficial wound infection x1
7	Sonmez, 2013 [2]	Unilateral MIS-TLIF with Pedical Screw Instrumentation	10 (60%)	Prospective	24	100	150	2,900 Turkish Lira	-78.82%	-55.07%	2.2	Unilateral MIS TLIF with PSI had comparable results to bilateral instrumentation in improving back pain and was much more cost-effective	NR	None
		Bilateral MIS-TLIF with Pedical Screw Instrumentation	10 (50%)			147	165	4,700 Turkish Lira	-79.76%	-50.68%	2.3		NR	None
8	Chen, 2009 [49]	TLIF	43	UTD	45 (24-72)	NR	NR	NR	+62.8% in JOA score	NR	NR	The mean JOA score was improved from 9.3 before surgery to 25.0 at the final follow-up visit. The fusion rate was 100% two years postoperatively. No implant failure	86.1% based on JOA score	Three patients (7%) had transient neurological deficits
9	Fu, 2005 [22]	Discectomy	23	Retrospective	88.7 (60 –134)	100.9 ± 22.8	162.7 ± 106.8	NR	+62.45% in JOA score	NR	4.7 ± 1.4	Intraoperative blood loss, length of surgery, and length of hospitalization were significantly less in patients undergoing discectomy alone than in patients with fusion.	78.3% based on JOA score	3 dural tears
		Discectomy with PLF	18			166.3 ± 26.7	546.7 ± 206.1	NR	+66.02% in JOA score	NR	6.2 ± 1.1		83.3% based on JOA score	1 superficial infection, 2 dural tears, and 3 residual donor site pain
10	Huang, 2002 [42]	PLIF with single, central cage and bilateral PSF	28 (64.3%)	Retrospective	14.4 (8-39)	NR	NR	NR	NR	NR	NR	Rate of bony fusion was 82.14%. Several patients with improved economic and functional status	92.86%	1 dural tear, 1 with transient paresthesias, and 1 with transient bladder atony

**Figure 1:**
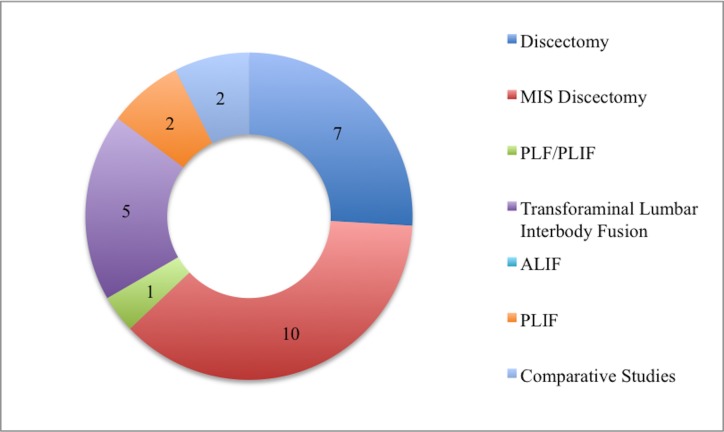
Number of Papers by Type of Surgery MIS: Minimally Invasive Surgery, PLF: Posterior Lumbar Fusion, PLIF: Posterior Lumbar Interbody Fusion, ALIF: Anterior Lumbar Interbody Fusion

**Figure 2:**
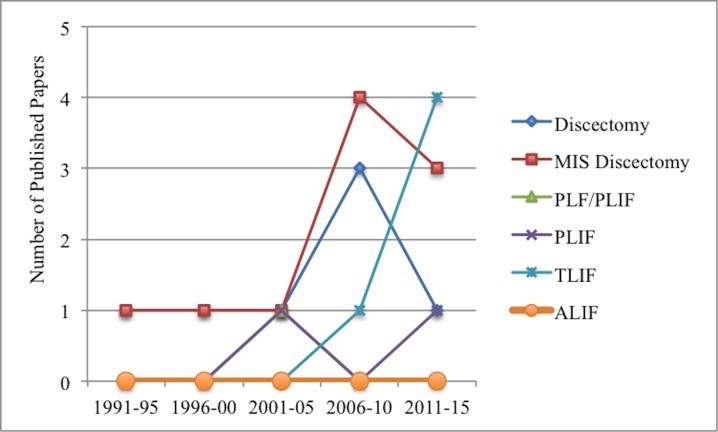
Number of Papers by Type of Surgery and Year of Publication MIS: Minimally Invasive Surgery, PLF: Posterior Lumbar Fusion, PLIF: Posterior Lumbar Interbody Fusion, TLIF: Transforaminal Lumbar Interbody Fusion, ALIF: Anterior Lumbar Interbody Fusion

Of the 27 articles reviewed for analysis, seven discussed outcomes from discectomy, 10 from a minimally invasive discectomy, five from TLIF, two from PLIF, one from both PLF/PLIF, and two comparative studies comparing discectomy and discectomy with fusion (Figures 1-2) [22, 27]. Six of the twenty-seven articles either had a follow-up time < 24 months or were not reported; the other 21 articles had at least a 24-month follow-up. There were seven prospective studies (26%), 19 retrospective studies (70%), and one where we were unable to determine whether it was a prospective or retrospective study.

### Discectomy

Of the nine articles reporting on the outcomes of discectomy for rDH, five (55.5%) reported VAS, JOA, or ODI scores for their study population. Of the five that did report these variables, four were on the JOA scale, which is a modified ODI [22, 27-29]. The percent improvement in JOA among these studies ranged from 52.17% to 64.8%. Only one study reported ODI scores [30]. As a result, no accurate calculations can be made to determine the average VAS and ODI changes after discectomy for rDH. Seven of the nine studies reported the percentage of patients showing good or excellent outcomes, which ranged from 70.60% to 89% [29, 31-32]. Of the total 326 patients undergoing discectomy from nine studies, dural tear was the most common complication reported occurring in 26 patients (8%) with three studies not reporting on complications. One reherniation occurred in 0.3% [27]. Neurological deficits or nerve root injuries occurred in five patients (1.5%). 

### Minimally invasive discectomy

Of the ten articles reporting on the outcomes of minimally invasive discectomy for rDH, six (60%) reported VAS and four (40%) reported ODI. Percent improvement in VAS among these studies ranged from 50.77% to 86.57%, indicating an overall pain reduction after operation for rDH using a minimally invasive discectomy. Percent improvement in ODI ranged from 61.32% to 88.56% (based on preoperative and postoperative values). Four of the nine studies reported the percentage of patients showing good or excellent outcomes, which ranged from 81% to 90.2% [23, 33-40]. Of the total 579 patients undergoing minimally invasive discectomy, dural tear was the most common complication reported and occurred in 23 patients (4%). Twelve patients had reherniation (2%). One of the 10 articles reviewed did not report on complications [38]. Neurological complications occurred in seven patients (1.2%): four with transient dysesthesia and three with nerve root irritations.

### Posterolateral fusion (PLF)

Three studies were noted to have evaluated patients undergoing PLF after rDH. One of these studies performed PLIF in 12 of the 14 patients evaluated, so it was instead excluded from this category [39]. The two remaining studies analyzed the PLF treatment in rDH and reported back pain based on the JOA scale, showing an improvement by 60.71% and 66.02% [22, 27]. El Shazly, et al. and Fu, et al. reported good or excellent outcomes in 86.70% and 83.3%, respectively. El Shazly, et al. also found PLF to be more cost effective than TLIF, but patients undergoing TLIF had a better improvement in JOA score (70% vs. 60.7%, respectively) and a larger percentage showing either good or excellent outcomes (93.3% vs. 86.7%). Fu, et al. found much longer OR times and larger EBL with PLF compared to discectomy alone but, overall, showed better outcomes with PLF. Of the 33 patients from the two studies who underwent PLF, three patients had dural tears (9%) and two had superficial wound infections (6%).

### Posterior lumbar interbody fusion (PLIF)

One of the three studies analyzed reported preoperative and postoperative VAS with a percent improvement of 46.02% [21]. Lequin, et al. reported 46% with good outcomes, Huang, et al. reported 92.86% with good or excellent outcomes, and Niu, et al. reported 93% with good or excellent outcomes [21, 41-42]. There were 68 patients who underwent PLIF between the three studies reviewed. Three patients had dural tears (4.4%) and six patients had neurological complications (8.8%). The neurological complications included worsening or new neurological deficits in four patients, one patient with transient paresthesias, and one patient with bladder atony.

### Transforaminal lumbar interbody fusion (TLIF)

Four of the six studies used the VAS metric to assess pain while the other two utilized the JOA scale. VAS improvement ranged from 54% to 86.5%. JOA scale change improvement was reported from 62.8% to 70%. Percent showing good or excellent outcomes ranged from 68.3% to 93.3% in the five studies reporting these findings. Niesche, et al. found no complications utilizing a minimally invasive TLIF approach with 85% showing good or excellent outcomes [43]. There were 216 patients who underwent TLIF from the six studies reviewed. Five patients (2.3%) had dural tears, 10 with neurological deficits postoperatively (4.6%), and three requiring revision surgery (1.3%). 

## Discussion

In reviewing the 23 articles that reported treatment outcomes for rDH, it is still difficult to ascertain which intervention is the most appropriate to use. All of the papers showed overall positive results in relieving pain when comparing preoperative and postoperative functional outcome measures, such as the VAS, JOA, and ODI. VAS and ODI are currently the most valuable resources of objective data in measuring the level of success. It is difficult, however, to identify any objective measures of success through radiographic imaging. A study by Cheng, et al. looked at the rate of first-time recurrent herniations in 207 patients based on the type of primary surgery and found that there was a lower rate of recurrence using a traditional open approach versus a microendoscopic discectomy or percutaneous endoscopic discectomy (37.8% vs. 47.1% and 70.6%, respectively) [44]. There is an insufficient amount of published data to help determine the most appropriate method of treating rDH at this time. Being that disc herniation is one of the most common back problems requiring surgical intervention, identifying the appropriate methods to accurately diagnose and treat rDH with standard outcomes measures would be worthwhile to investigate. This would also help to establish the most cost-effective intervention (surgical and non-surgical) with the lowest associated morbidity.

The choice between repeat discectomy and discectomy with fusion for rDH has been a highly debated topic [45-49]. In one perspective, fusion is usually costlier, associated with more complications, longer OR times, larger EBL, and longer hospitalizations. In the analysis performed here, it seems that TLIF is the more superior fusion option based on the greatest decrease in VAS/ODI compared to the other fusion studies reviewed. However, the lack of published data on other forms of fusion and limited comparative studies makes it more difficult to accurately make this conclusion. One of the two comparative studies reviewed by Fu, et al. compared discectomy and discectomy with PLF and found better improvement of pain after fusion. However, fusion was also associated with more complications, more blood loss, and longer operative times compared with discectomy alone [22].

From a surgical decision-making perspective, it was difficult to determine indications or a reliable algorithm for selection of fusion for rDH from the articles reviewed. Mroz, et al. published their findings from a survey identifying the surgical treatment patterns among spine surgeons in the United States for lumbar rDH and found that the number of surgeries performed and years of practice had a statistically significant impact on the type of surgery performed [20]. They concluded that a surgeon practicing for 15-20 years is less likely to perform a revision microdiscectomy with fusion versus revision microdiscectomy alone. However, they also found that higher volume surgeons with > 200 cases per year were more likely to perform a fusion to address rDH. This variance could be indicative of multiple factors, including surgeon preference and patient characteristics, but we need to consider the lack of proper evidence-based data as a probable reason for the lack of definitive recommendations. One consideration is to utilize the National Neurosurgery Quality and Outcomes Database (N^2^QOD) registry, which is a prospectively collected sampling of patients who experienced same-level, same-side rDH, had either a discectomy or arthrodesis, and had one-year follow-up [50]. This registry collects the same data variables on all patients, which allows for better statistical analysis than when trying to combine data in a meta-analysis. Additionally, this will assist in performing more accurate comparative analyses to determine indications or generate a reliable algorithm for the treatment of rDH. The abstract by McGirt, et al. found greater healthcare utilization and morbidity with arthrodesis in their comparative analysis of 417 patients in the N2QOD registry and concluded that revision discectomy is the more efficient treatment option [50].

In regards to reporting the rates of reherniation, one concern in the literature is the lack of distinguishability between radiographic evidence of reherniation and symptomatic reherniation. Lebow, et al. found that about one-fourth of patients who underwent a lumbar discectomy had radiographic evidence of reherniation with the majority being asymptomatic [51]. Furthermore, these asymptomatic reherniations did not develop any clinical consequences at the two-year follow-up. In regards to the studies reviewed in this analysis, it is unclear whether they had radiographic or symptomatic evidence of reherniation. For example, Vik, et al. reported outcomes on 39 patients who underwent revision surgery due to suspected herniation but then found that recurrence had been found in only 14 of them [52]. Similarly, Ozgen, et al. studied 114 patients with previous lumbar disc surgery who underwent re-exploration and found that only 56 had a true recurrence of herniation [18]. Epidural fibrosis, a major intraoperative finding in non-rDH revision surgeries, is often difficult to distinguish with advanced imaging and presents with similar clinical symptomology. This has been shown in previously published studies to be associated with poor results from revision surgical intervention [53-57]. It appears from these reported data that many patients who do not have a true recurrence are still undergoing surgical treatment in place of a more conservative management without the morbidity of a second operation. Formulating a more concrete set of diagnostic criteria for rDH would help delineate the use of symptomatic versus radiographic diagnosis. It would be worthwhile to perform a comparative analysis along with a cost-effectiveness analysis to determine if the costs of imaging to diagnose rDH outweigh the costs of unnecessary operations for patients who were incorrectly diagnosed or having clinical symptomatology alone.

In our review of the literature for the cervical and thoracic spine, the rates of rDH were rarely mentioned. Although the incidence of rDH in these spinal regions occurs less frequently compared to the lumbar spine, the management is somewhat similar. It would be of value to determine the efficacy of these various interventions to better guide our treatment algorithms.

### Study limitations

Some of the limitations of this study include the small number of papers currently published on the treatment of rDH and the reporting of standardized outcome measures. Additionally, of the papers that were included, there was a broad spectrum of definitions of rDH, making it difficult to compare the patients selected for treatment and their outcomes. The lack of uniformity in postoperative data collection was further amplified by not all of the studies reporting similar time points after surgery for postoperative VAS and ODI. The possible variability in when the VAS and ODI were recorded in each paper could be a limitation that we were unable to correct for, given the data reported.

### Future outlook/recommendations

Future studies assessing outcomes of the treatment of recurrent disc herniation are needed in order to establish a better perspective on the proper approach to and management of recurrent disc herniation. Studies using registries can help better elucidate these questions by allowing more comparative analyses to be done and work towards making more accurate treatment recommendations and algorithms [45]. This includes further investigation of risk factors for recurrence and comparative studies on the outcomes of these surgical techniques. Identifying true risk factors for recurrent herniation can help stratify patients for different treatment options and possibly have an impact on costs if reherniation can be avoided. Another consideration is the question of accurate versus precise diagnosis of recurrence. Although it is difficult right now to establish an accurate diagnosis, having a better definition of rDH would allow for better precision and standardization of what the literature describes as rDH. Several of the studies reviewed noted performing MRIs on each patient to determine if reherniation had occurred, but this may not be necessary or the most cost-effective method of diagnosis and treatment.

We developed a set of recommendations for future studies on surgical outcomes, which are summarized in Table 3. In order to achieve more accurate results on the outcomes of a surgical intervention for rDH, prospective studies with a minimum two-year follow-up are needed to properly assess the long-term implications after surgery. We hope that these factors, along with already published reporting guidelines, will help produce studies that can change the way patients are treated for rDH in the future.

**Table 3:** Recommendations for Future Studies in Recurrent Disc Herniation Treatment.

Recommendations:
1. How recurrence of disc herniation was determined (imaging, symptomatology, etc.)
2. Which level and side (ipsilateral or contralateral) the reherniation was located
3. Time frame after primary operation
4. Which intervention(s) are being studied
4. Reporting of preoperative VAS/ODI
5. Reporting postoperative VAS/ODI immediately after surgery and at 6-month intervals for at least 2 years
6. Percent with good or excellent outcomes using MacNab's assessment
7. Complicating factors to reherniation (i.e. fibrosis, etc.)
8. Time until return to work or regular daily activities

## Conclusions

The current analysis was not able to conclude on any significant difference in outcomes in comparing one surgical method to another. This is largely based on the lack of standardized reporting of outcomes in the literature, which makes it difficult to combine these data points for analysis with such a small power. However, in reviewing the few selected articles that met our stringent criteria, we concluded that fusion may have a greater improvement in pain and functional outcomes compared to reoperation without fusion at the cost of more complications, increased blood loss, and longer operative times for the treatment of rDH.
